# Variability in Background Urinary Concentrations of the Hydrogen Sulfide Biomarker Thiosulfate

**DOI:** 10.1021/acsomega.2c04112

**Published:** 2022-10-19

**Authors:** Bassam Lajin

**Affiliations:** Institute of Chemistry, Analytical Chemistry for the Health and Environment, University of Graz, Universitaetsplatz 1, Graz 8010, Austria; Institute of Chemistry, ChromICP, University of Graz, Universitaetsplatz 1, Graz 8010, Austria

## Abstract

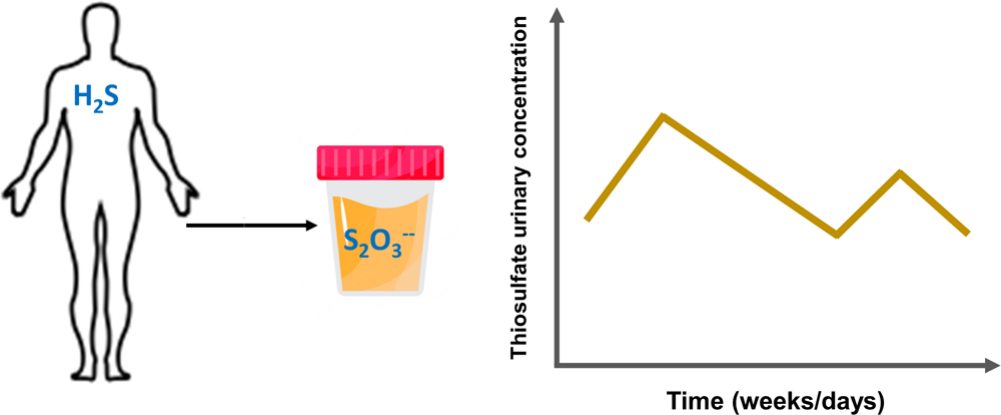

Hydrogen sulfide is a toxic gas at high concentrations but has recently attracted attention as a naturally produced gaseous signaling molecule in various tissues of the human body, playing key physiological roles at low nanomolar concentrations. This has wide implications for chronic exposure to this gas in air at low levels far below toxicity. Thiosulfate is the currently used biomarker for exposure to hydrogen sulfide via inhalation but has been mainly employed for acute exposure. It is unknown how background thiosulfate concentrations vary on an intraindividual and interindividual basis in humans at normal ambient hydrogen sulfide levels (<1 μg m^–3^), which is key for the interpretation of its levels as biomarker for low-level hydrogen sulfide exposure. In the current work, the variability in thiosulfate urinary excretion in a total of 168 urine samples collected from eight volunteers over a period of 8 weeks was investigated. The determination of thiosulfate in urine was carried out by UHPLC-MS/MS. The total average concentration ± SD was 16 ± 6 μM (*n* = 168). Average urinary thiosulfate concentrations in the studied volunteers were within the range of 10–20 μM, but it was found that urinary thiosulfate can show significant day-to-day and week-to-week variability in some individuals (up to 10-fold), despite adjusting for urine specific gravity. In light of the presented variability data and previous studies about the lack of consistent response of thiosulfate to low levels of hydrogen sulfide inhalation exposure, and based on a review of the biochemistry of the production of thiosulfate and its various biological sources, it can be argued that thiosulfate might not be suitable as a biomarker for chronic environmental exposure to low levels of hydrogen sulfide via inhalation.

## 1 Introduction

Hydrogen sulfide is a naturally occurring gas that was involved in the development of some of the earliest forms of life on earth billions of years ago by serving as a source of energy in anoxygenic photosynthesis.^1,2^ It has also been suggested that hydrogen sulfide might have played a role as a transitional electron donor in the evolution of oxygenic photosynthesis, which led to dramatic changes in earth’s atmosphere, referred to as the great oxygenation event that occurred about 2.3 billion years ago.^3^ The continuous rise in atmospheric oxygen levels led to the oxidation of hydrogen sulfide and a sharp decrease in its atmospheric levels, which was followed by the evolution of animals and land plants,^3^ and thereby the role of hydrogen sulfide drastically shifted, as this gas turned from being a primary and essential source of energy essential to some of the earliest life forms on our planet into a highly toxic gas lethal to most forms of life in current existence.

Sulfur gases in the atmosphere of today’s earth originate from natural as well as anthropogenic sources. Natural emission of sulfur-containing gases is estimated at 52 Tg per year, but this includes mainly sulfur dioxide and dimethylsulfide and only about 4.4 Tg per year as hydrogen sulfide.^4^ The major source of natural hydrogen sulfide is sulfate-reducing anaerobic bacteria.^5^ Hydrogen sulfide is also a natural component in natural and volcanic gases, hot springs, and unrefined petroleum. Although anthropogenic sulfur-containing gas emission (70–100 Tg per year^6,7^) exceeds natural emission, most anthropogenic sulfur is emitted as sulfur dioxide, and only about 3 Tg per year is emitted as hydrogen sulfide,^6,7^ primarily originating from livestock production and industrial processes such as pulp and oil refinement.^8^

Although primarily regarded as a toxic gas, hydrogen sulfide has been recently a subject of thorough investigation as a naturally and endogenously produced gaseous signaling molecule in humans, playing a variety of essential biochemical roles.^9,10^ The participation of hydrogen sulfide in normal biological processes at low steady-state tissue concentrations of <0.1 μM^11,12^ suggests that exposure to hydrogen sulfide in ambient air even at low subtoxic levels can have significant effects on human health, which have been frequently explored.^13,14^ Chronic exposure to hydrogen sulfide is not only relevant under occupational settings but also for human populations living in close proximity to geothermal power plants, which are gaining increasing importance as an alternative source of energy.^15^ Indeed, hydrogen sulfide concentrations in air >50 μg m^–3^, which constitutes an elevation by >100-fold above normal ambient levels, were reported at >25 km distance away from geothermal power plants,^16−18^ and the health aspects associated with this level of exposure to hydrogen sulfide have been a recurrent source of debate in relation to the overall safety of geothermal energy.^19,20^

Reliable measurement of hydrogen sulfide to investigate human exposure to this gas is challenging due to its high volatility particularly under the neutral to weakly acidic pH values encountered in biological samples. Thiosulfate can be produced in the human body from hydrogen sulfide through a set of enzymatic processes,^21^ and its levels in bodily fluids have been widely utilized to indicate acute exposure to hydrogen sulfide.^22−24^ However, apart from a small-scale investigation provided by Durand and Weinstein,^25^ the utilization of this biomarker under conditions of chronic low-level exposure to hydrogen sulfide has been largely underexplored.

The interpretation of thiosulfate levels in humans and its correlation with hydrogen sulfide exposure, particularly at low levels, must be based on data involving the intraindividual and interindividual variability in its background concentrations in human matrices, which is currently lacking. The aim of the present work was to investigate the variability in thiosulfate urinary excretion in a group of healthy volunteers living in the nongeothermal city of Graz under conditions of normal ambient exposure (<1.0 μg m^–3^) and discuss the general reliability of thiosulfate as a biomarker for hydrogen sulfide and its applicability under conditions of low-level exposure in the light of the presented data as well as the available information with regard to the biochemistry of hydrogen sulfide.

## 2 Experimental Section

Urine collection was performed as previously described.^26^ Briefly, a total of eight volunteers were included in the study (three females and five males; age range, mean, and SD 18–60, 37, and 13 years, respectively). Consent was obtained from the volunteers who were co-workers at the university of Graz, Austria and the study was approved by the ethical committee at the university of Graz (GZ: 39/46/63). Volunteers were asked to collect urine on Corning polypropylene 300 mL sample collection containers (Corning, NY, USA), in the morning and evening for seven consecutive days followed by a morning urine sample on a weekly basis for the next 7 weeks. Portions of the samples were transferred to 5 mL Eppendorf tubes (Eppendorf, Vienna, Austria) and stored at −80 °C until analysis.

Determination of thiosulfate was performed using the method that we developed and analytically validated as previously described,^27^ using an Agilent 1260 Infinity II LC system (Agilent Technologies, Waldbronn, Germany) including a reversed phase chromatographic column Zorbax Eclipse Plus C18 RRHD column (50 mm × 2.1 mm, 1.8 μm particle size, Agilent Technologies, Waldbronn, Germany) connected with a tandem molecular mass spectrometric system (Agilent triple quadrupole Ultivo LC/TQ) for detection.

Urine was filtered through Nylon-66 syringe filter (pore size: 0.22 μm, BGB Analytik GmbH, Germany) into 0.7 mL polypropylene HPLC vials, before injection onto the chromatographic column. Thiosulfate-^34^S was synthesized in-house as previously described^27^ and employed as an isotopically labeled internal standard at a concentration of 20 μM by coinjection with the urine samples in order to compensate for matrix effects. For mobile phase preparation, water obtained from a purification system (Millipore GmbH, Vienna, Austria) and methanol of HPLC grade (HiPerSolv CHROMONORM, Germany) were used. To achieve retention on the reversed-phase column, a member of a newly introduced generation of cationic ion-pairing reagents (heptafluorobutylamine) with desirable properties for mass spectrometric detection^28^ (heptafluorobutylamine, Manchester organics, Manchester, UK) was incorporated at a concentration of 0.2% v/v in a mobile phase containing 10% methanol for isocratic separation at a flow rate of 0.4 mL min^–1^. Thiosulfate and Thiosulfate-^34^S were detected by monitoring the mass transitions 113 → 80 and 117 → 82, respectively. Further information about the analytical method including chromatographic separation as well as detailed chromatographic and mass spectrometric conditions can be found in previous work.^27^

To account for variability in fluid intake, we normalized concentrations according to specific gravity determined with a Leica TS 400 total solids refractometer (Leica Microsystems, Buffalo, NY, USA) using the equation *C*_norm_ = ((SG_mean_ – 1)/(SG_sample_ – 1))*C*_sample_, where *C* denotes a concentration and SG denotes specific gravity.

## 3 Results and Discussion

The mean ± SD urinary thiosulfate concentration in all samples investigated (*n* = 168) from the eight volunteers was 16 ± 6 μM. These concentrations are in general agreement with previously reported values for background urinary thiosulfate concentration in individuals unexposed to hydrogen sulfide (e.g., 31 ± 16 μM (*n* = 5)^29^ and 22 ± 17 μM (*n* = 12)^30^).

**Figure 1:**
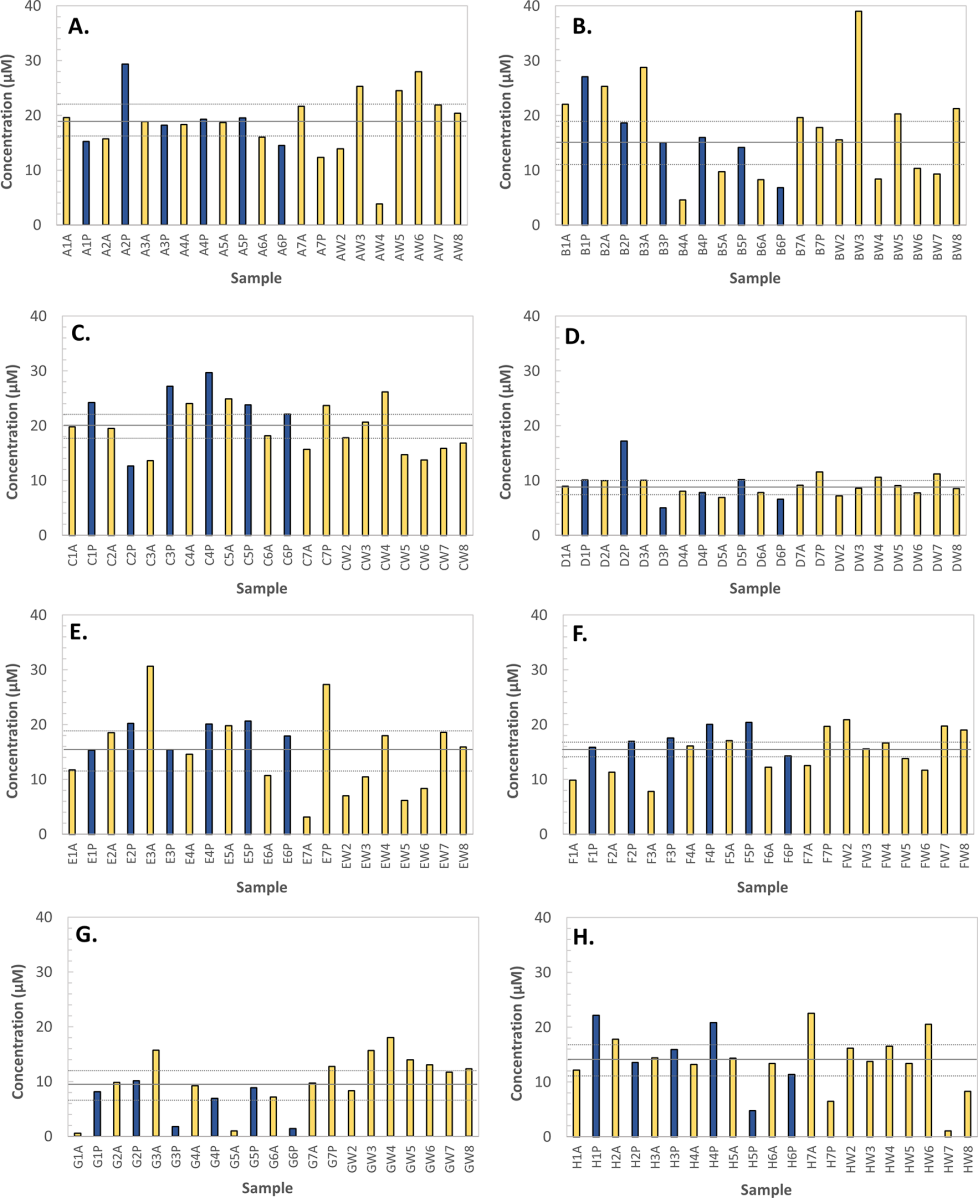
Urinary levels of thiosulfate in the eight studied volunteers. The graphs show concentrations normalized to specific gravity in volunteers A–H. For each volunteer one morning urine sample (e.g., A1A, A2A, etc.) and one evening urine sample (e.g., A1P, A2P, etc.) were collected over 7 days. After the first week, one morning urine sample on a weekly basis was collected (AW2, AW3, etc.) for the next 7 weeks. Morning and evening samples were indicated with yellow and blue, respectively. The solid line indicates the average concentration and the dotted lines indicate the 95% confidence interval.

Although the average concentrations across the investigated volunteers were within a relatively narrow range (10–20 μM), there was considerable intraindividual variability (up to 10-fold) in some volunteers despite adjusting for specific gravity (Figure 1). Furthermore, the extent of the intraindividual variability appeared to differ among the different volunteers (e.g., compare volunteers B, G, and E with volunteers D and F).

An overview of the biochemistry of the production of thiosulfate can indicate possible sources for the observed intraindividual variability in its urinary excretion. Thiosulfate is produced from hydrogen sulfide via a multistep mitochondrial enzymatic pathway.^21^ Notably, sulfite is reported to be an intermediate that is converted to thiosulfate in the last biosynthesis step via a sulfur transferase enzyme (rhodanese).^21^ Sulfite can originate from the diet as it is widely used as an additive, particularly in common alcoholic beverages such as beer and wine, which can contain concentrations in excess of 100 mg L^–1^.^31^ It is therefore plausible that dietary sources of sulfite may influence thiosulfate levels in urine. The effects of a sulfite-rich diet on thiosulfate urinary excretion have not been previously investigated.

Hydrogen sulfide is produced endogenously from cysteine in mammalian tissues at rates reported within the range of 10–20 μmol h^–1^ kg tissue^–1^,^12^ and the steady-state concentrations of hydrogen sulfide were reported to be ca. 15 nM in mouse brain and liver.^12^ Most hydrogen sulfide in the human body, however, originates from the gut bacteria, as concentrations of hydrogen sulfide within the range 1.0–2.4 mM were reported in the contents of the large intestine,^32^ where oxidation to thiosulfate by the heavily expressed rhodanese enzyme in the colonic mucosa^33^ is thought to be the primary defense mechanism against buildup of toxic levels of hydrogen sulfide.^34^ Therefore, urinary thiosulfate would be expected to significantly respond to dietary and physiological changes that alter the human gut microbiome, which is yet another source of variability in the production of thiosulfate that remains unexplored.

An overview of the enzyme/gene expression profile in human tissues reveals that the enzymatic activities leading to thiosulfate production are highest in the liver and gastrointestinal tract and low in the respiratory tract.^33^ Indeed, elevation of urinary thiosulfate even following acute exposure to inhaled hydrogen sulfide is reported to be rather inconsistent,^35^ particularly in fatal cases where it is assumed that the transport of thiosulfate via blood to target tissues such as the liver where it can be metabolized to thiosulfate may not be rapid enough to result in significant elevation in urinary thiosulfate levels that can be utilized as evidence for hydrogen sulfide poisoning.^23^ Furthermore, Durand & Weinstein reported only a marginal increase (from 7.2 to 9.8 μmol mol^–1^ creatinine) in the average urinary thiosulfate concentration in a group of eight volunteers following exposure to hydrogen sulfide even at levels >1000-fold higher (1.0–10 mg m^–3^) than normal levels of hydrogen sulfide in ambient air (<1.0 μg m^–3^).^25^

In light of the presented data on the variable background concentrations of thiosulfate in urine as well as the observed inconsistency in its response to hydrogen sulfide exposure in previous studies, which can be rationalized by the above explained factors regarding the mechanism of production of this metabolite and its numerous possible sources of variability, the reliability of urinary thiosulfate as a biomarker for environmental exposure to inhaled hydrogen sulfide, particularly at low levels, can be questionable. The need arises for new biomarkers that are more selective for hydrogen sulfide exposure through the lungs, which will be the subject of our future work.

## Notes

All data relevant to this study is included in the main manuscript.

## Dedication

This work is dedicated to my supervisor Kevin Francesconi, whose support in a journey out of a war-torn country provided new career hopes.
